# Preparing for a community-based agriculture-to-nutrition trial in rural Malawi: formative research to assess feasibility and inform design and implementation decisions

**DOI:** 10.1186/s40814-021-00877-1

**Published:** 2021-07-07

**Authors:** Gabriella Chiutsi-Phiri, Alexander A. Kalimbira, Leonard Banda, Patson C. Nalivata, Marion Sanuka, Zione Kalumikiza, Limbanazo Matandika, Joseph Mfutso-Bengo, Elizabeth Allen, Elaine Ferguson, Joanna Sturgess, Martin R. Broadley, Simon Langley-Evans, Kate Millar, Dawd Gashu, Edward J. M. Joy

**Affiliations:** 1 Lilongwe University of Agriculture and Natural Resources, PO BOX 143, Natural Resources College, Lilongwe, Malawi; 2 Lilongwe University of Agriculture and Natural Resources, PO BOX 219, Bunda College, Lilongwe, Malawi; 3 School of Public Health and Family Medicine, College of Medicine, University of Malawi, Blantyre, Malawi; 4 Faculty of Epidemiology and Population Health, London School of Hygiene & Tropical Medicine, London, WC1E 7HT UK; 5 School of Biosciences, University of Nottingham, Sutton Bonington Campus, Loughborough, LE12 5RD UK; 6 Center for Food Science and Nutrition, Addis Ababa University, Addis Ababa, Ethiopia

**Keywords:** Biofortification, Community-based trials, Formative research, Community sensitisation

## Abstract

**Background:**

This study reports findings from formative research conducted to assess the feasibility and inform the design and implementation of the Addressing Hidden Hunger with Agronomy (AHHA) trial. The AHHA trial was a randomised, controlled trial conducted in rural Malawi, in which participants were given maize flour biofortified with selenium or control flour not biofortified with selenium for a period of 10 weeks, after which blood samples were taken to measure selenium status.

**Methods:**

Formative research was conducted in villages near to the AHHA trial study site 1 year before the planned intervention. A short questionnaire with adult women (n = 50), focus group discussions with male (n groups = 3) and female (n groups = 3) community members, and in-depth key informant interviews (n = 7) were conducted to understand community practices and perceptions.

**Findings:**

Meals were typically cooked and eaten at home in this community, while participants reported that maize flour would be less readily sold than maize grain – important considerations for the design of the trial. Regarding intervention delivery, we identified potential concerns around effects on fertility, links between blood sampling and witchcraft, and the potential for social stigma if community members considered participants lazy for receiving free flour. Participants reported that involvement of the Malawi government partners including health extension workers would increase trust.

**Interpretation:**

Following the formative research, the AHHA trial appeared feasible. However, community sensitisation would be essential to address potential fears and concerns; effective sensitisation would support recruitment and treatment adherence, and would protect the safety and wellbeing of participants and researchers. People in positions of authority and trust including village headmen, religious leaders, health and agriculture extension workers, and community care groups should be involved in community sensitisation.

**Supplementary Information:**

The online version contains supplementary material available at 10.1186/s40814-021-00877-1.

## Key messages regarding feasibility

- *What uncertainties existed regarding the feasibility?*

Would participant households in the AHHA trial be willing to receive and consume the trial maize flour in place of their own flour? How could the trial team improve acceptability and reduce the risk of households sharing or selling the trial flour? Would participants be willing to provide blood samples?

- *What are the key feasibility findings?*

Households would be willing to receive and consume the trial flour in place of their own, and limiting sharing with other households is also feasible. However, clear communication of the importance of consuming the flour within the household will be required. Also, community sensitisation will be required to improve acceptability and address potential fears, concerns and rumours regarding consumption of the flour and provision of blood samples.

- *What are the implications of the feasibility findings for the design of the main study?*

A trial design with households randomised to treatment (rather than a cluster design) was considered feasible. Extensive community sensitisation would be required to ensure the acceptability of the trial intervention and blood sampling. The sensitisation process should involve influential and trusted members of the community including health extension workers and village head men. Members of the community should also be invited to see the maize under production and processing, to increase trust in the flour.

## Introduction

### Background

Formative research aids in the design and delivery of successful trials. Formative research is particularly valuable where the intervention is complex or is implemented in a challenging context [1, 4, 8, 14]. This study reports the findings from formative research conducted in preparation for a community-based trial that was successfully carried out in rural Malawi—the *Addressing Hidden Hunger with Agronomy* (*AHHA*) trial [10]. The AHHA trial aimed to determine the efficacy of addressing selenium deficiency in rural Malawi through consumption of agronomically biofortified maize flour. Agronomic biofortification is the process of enriching edible crops through application of micronutrient fertilizers [16].

#### Selenium deficiency in Malawi

Micronutrient deficiencies, also known as ‘hidden hunger’, are widespread in Malawi. Selenium is an essential micronutrient for human nutrition [9, 17]. In a recent Malawian national survey, 63% of women of reproductive age had suboptimal plasma selenium concentrations [15]. Deficiency of selenium may impair thyroid and antioxidant function, and cognitive development in children [5, 7]. Agronomic biofortification may be a cost-effective strategy to address selenium deficiency in Malawi [3, 11]; however, there is a global evidence gap in the efficacy and effectiveness of agronomic biofortification for alleviating micronutrient deficiencies [6].

#### The AHHA trial and formative research

The AHHA trial was a pragmatic, community-based trial, in which 180 women of reproductive age and 180 school-aged children were recruited, and households were randomised to one of two arms [10]. One arm received maize flour enriched with selenium (intervention), while the other received maize flour not enriched with selenium (control), with flour distributed at centralised points in participant villages for 10 weeks. The primary outcome was selenium status measured in blood serum, and blood samples were taken from participants at baseline and endline. The trial was successfully implemented in rural Kasungu District, Central Region, Malawi, with high recruitment rates and adherence and low loss to follow-up.

Twelve months prior to the AHHA trial intervention, formative research was conducted nearby to the trial study area to assess the feasibility of the trial. The current paper reports the findings of this formative research and how this informed the design and implementation of the trial.

## Methods

### Prior identification of risks

Prior to the formative research, a trial design was proposed in which participant households were randomly allocated to treatments, receiving either intervention or control maize flour. The trial study team identified a set of questions that needed to be answered to confirm that the trial design was feasible and to inform trial delivery:

1. Should maize be distributed as grain or flour? If flour, should it be whole grain (*mgaiwa*), partially refined (*granmill*), fully refined (*woyera*), or composed mainly of maize husks (*madea*)?
2. Would participants and their household members be willing to consume distributed maize in place of their own? What steps should be taken to increase acceptability and adherence?
3. Would distributed maize be consumed or would it be sold/traded?
4. To what extent do households share maize, and would this substantially affect the exposure to the biofortified flour or lead to contamination between treatment arms? What steps could be taken to reduce inter-household sharing?
5. Would participants be willing to provide blood samples? What steps should be taken to increase acceptability?
6. Would participants face stigma from other community members or from those outside the study area, for example due to receiving free maize flour or due to providing blood samples?
7. Which community members should be involved in supporting the trial, in representing the community, and in raising or addressing community concerns? How should these community members be involved?

### Study area

The formative research took place among five villages in a rural community in Chambwe sub-Traditional Authority in Chilowamatambe Traditional Authority (TA), in Kasungu District in the Central Region of Malawi. Chambwe sub-TA is in the same extension planning area (EPA) as Wimbe TA where the AHHA trial was conducted. The EPA is the most decentralised office through which agricultural extension services are provided. A list of villages (n = 96) in the study area was obtained from the EPA from which five villages were randomly selected. The area is characterised predominantly by subsistence farming, alongside smallholder and estate tobacco production. The formative research was conducted approximately 30 km from the proposed AHHA trial study area, providing a relevant population while avoiding influencing potential trial participants.

### Study design

Formative research was conducted 12 months prior to the AHHA trial start date. Mixed methods research was chosen to provide a deeper understanding of community practices and perceptions of the proposed trial, including any concerns participants would have with consuming the trial flour or providing their blood samples. A short questionnaire was conducted with adult women, six focus group discussions (FGDs) were conducted with adult men (n groups = 3) and adult women (n groups = 3), and seven in-depth key informant interviews (KIIs) were conducted with influential members of the community. The KIIs provided another perspective on the potential challenges of delivering the trial and helped the research team identify strategies to resolve these challenges.

### Eligibility

Inclusion criteria were as follows:

Questionnaires:

- Adult women aged 20–45 years
- Residents of the study area
- Non-pregnant (self-reported)
- Willing and able to provide consent

FGDs:

- Adult men or women aged 20–45 years
- Residents of the study area
- Willing and able to provide consent

KIIs:

- Adult males or females who held relevant influential roles and responsibilities in the community and who consented to participate

Exclusion criteria were as follows:

FGDs:

- Community leaders

Inclusion criteria for questionnaire participants were similar to those subsequently used for the AHHA trial, which would assess the efficacy of consuming biofortified maize flour for alleviating selenium deficiency in adult women and children. Focus group discussions were conducted with women and men since we recognised that support for the trial, including willingness to receive and consume flour, would be required from all adults within a household and in the wider community. Community leaders were excluded from FGDs to avoid imbalances of social status that might make other participants reticent to speak up.

### Recruitment

For questionnaires, 50 participants were randomly recruited from the five participating villages, with 10 participants *per* village. The FGDs were conducted in three of the five villages only, with two FGDs *per* village—one with male participants and the other with female participants. Villages were selected on a convenience basis following guidance from the local agricultural extension office. Each focus group consisted of 8 participants. Separation by gender was considered appropriate in this context, where responses around willingness to consume the trial flour, provision of blood samples and trust in community members were considered likely to differ by gender. To sample participants within villages, the agricultural extension worker communicated with the village headmen who called residents to meet at a central place within the village. The research team briefed the community members on the objectives of the formative research and on the random selection process for participant recruitment. The random sampling was conducted publicly to ensure that community members understand that there was no bias in selecting participants. As such, the researchers prepared small pieces of paper, some with numbers. The papers were rolled up such that the numbers were not visible. The papers were tossed in a plate and the community members were asked to pick one paper from the plate without replacement. Individuals who picked a paper with a number were selected for FGDs. The exercise was repeated to select participants for questionnaires.

Interviewees for the KIIs were identified by the study researchers based on their experience working in the area, and subsequently through snowball sampling based on recommendations made by KII participants. The key informants (KIs) were a district nutrition officer, a district information officer, agriculture and health extension workers, a village chief, a primary school head teacher and a non-governmental organisation (NGO) officer involved in nutrition and food security activities in the district. The village headman was resident in the study area, while others were individuals who work with the community in their professional roles.

### Tool development

The research team initially developed the questionnaire and FGD guide in English. The tools were then translated to Chichewa and then back-translated to English by an independent translator to confirm its meaning was the same as the original (Additional Files 1 and 2). The Chichewa tools were subsequently piloted and refined before use in the field. The KII questionnaire was developed in English and translated to Chichewa by the research team (Additional File 3).

### Data collection

Short questionnaires were conducted with adult women in participants’ households by trained research assistants using electronic data capture on tablets via Open Data Kit [2]. Each questionnaire took approximately 30 min to complete.

The FGDs were conducted in locations free from distractions, such as church buildings or school blocks. Separate FGDs were held with adult men (n groups = 3) and women (n groups = 3). The FGDs, which lasted between 35 and 65 min, were facilitated by one Research Assistant, while another observed and took notes on themes emerging from the FGDs. The facilitator and observer had experience in conducting qualitative research including FGD facilitation. Both had completed training prior to the fieldwork. The FGDs were audio-recorded.

In-depth KIIs (n = 7) were conducted by study investigators in English or Chichewa, depending on the preference of the participant. Interviews were conducted in a location free from distractions and took approximately 40 min to complete. Interviews were audio-recorded and later transcribed.

The questionnaire and FGD data collection happened in parallel over a period of 3 days. The KIIs were conducted sequentially, with informants identified and recruited based on findings from the FGDs and from previous KIIs, and were conducted over a period of 5 days.

### Data analysis

The questionnaire data were analysed using descriptive statistical analysis in STATA (version 15; StataCorp, TX, USA). The FGDs (all in Chichewa) and KIIs (six in English, one in Chichewa) were transcribed verbatim soon after data collection. Chichewa transcripts were translated into English by study authors (GCP, MS). Thematic analysis of transcripts was conducted in MAXQDA (version 18; VERBI Software GmbH, Berlin, Germany) using a deductive approach with themes linked to the pre-identified trial risks (see above). Coding was done using a code tree. The findings from men and women were presented separately. Quotes were drawn from FGDs and KIIs to support the interpretation of findings.

### Ethics

Written consent was obtained from all participants prior to recruitment. Participants were provided with a printed participant information sheet in English or Chichewa, depending on their preference, and this was read aloud to ensure comprehension. Participants were asked to complete the consent form upon voluntary agreement to participate in the study. For illiterate participants, participants completed the informed consent sheet by using a thumb print with a signature provided by a witness. The witnesses were literate adults from the study community and independent of the study researchers. Witnesses were present during the whole informed consent process. Consent forms, data forms and transcripts were labelled with unique, anonymous participant IDs. Participants were compensated for their time with mobile phone vouchers at a value of MWK500 per participant. Prior to commencing any data collection, the facilitator reminded the participants of the following: purpose of the research, the expected duration of the questionnaire/FGD/interview, that FGD sessions/interviews would be audio-recorded, and that participants would receive the same compensation for their time even if they left the study at any point. All participants were asked to confirm that they were happy to proceed.

Participants were not coerced to engage in the study activities, and they were assured that they would not face any disadvantages if they withdrew their participation in the study at any time. Participant ID codes were used to maintain anonymity, and potentially identifying information was excluded from transcripts. Confidentiality of the data and the privacy of participants were respected at all times through use of passcode-protected tablets for data collection, and encryption and transfer of data from tablets to secure cloud storage on a daily basis. The study was reviewed and approved by research ethics committees at the College of Medicine, University of Malawi (reference P.05/18/2393) and the London School of Hygiene & Tropical Medicine, UK (reference 15730).

## Results

### Questionnaire findings

A total of 50 questionnaires were completed with 10 per village. The responses to questions 1 to 4 (Additional File 1) were used in the design of dietary data collection tools and are not reported here.

In terms of maize flour type, 35 households (70%) were planning to consume *woyera* (refined flour), 14 (28%) were planning to consume *granmill* (partially refined flour), 10 (20%) were planning to consume *mgaiwa* (whole meal flour) and zero households planning to consume *madea* (maize bran) in the following month. However, when participants were asked hypothetically which type of maize flour they would most like to receive as a gift, more selected *granmill* (52%) than *woyera* (36%). Potential reasons are explored in the FGD findings. *Mgaiwa* and *madea* were less popular (Table 1).

**Table 1:** Number (%) of participants reporting mgaiwa, granmill, woyera and madea preparations of maize flour as their preference, were they to receive this as a gift. Mgaiwa flour is unrefined, granmill is partly refined (bran removed), woyera is fully refined and madea is bran only

	***Mgaiwa***	***Granmill***	***Woyera***	***Madea***
1 (most preferred)	6 (12)	26 (52)	18 (36)	0 (0)
2	20 (40)	15 (30)	15 (30)	0 (0)
3	23 (46)	8 (16)	16 (32)	3 (6)
4 (least preferred)	1 (2)	1 (2)	1 (2)	47 (94)

In the questionnaire, 8 out of 50 participants reported eating *nsima* outside the household the previous day, of whom two reported it was at church gatherings, while others did not report a special occasion. Five participants gave away flour, and 6 participants received flour as a gift (Table 2).

**Table 2:** Among questionnaire participants (n = 50), the number that consumed *nsima* outside the home, received a gift of maize flour or gave away maize flour on the previous day

	Consumed ***nsima*** outside household	Received maize flour gift	Gave away maize flour
Did not occur	42	44	45
Occurred: with household in the village	5	3	2
Occurred: with household outside the village	2	3	3
Occurred: restaurant	1	0	0

The estimated amounts of flour given away or received as a gift ranged from < 1 kg to > 20 kg and 1 kg to > 20 kg, respectively. Nevertheless, the questionnaire results also indicated that the exchange of flour occurred with similar frequency with a household outside the village as within it. Similarly, for 3 out of 8 cases where meals were consumed outside the household, this occurred outside the village or at a restaurant. Thus, a cluster design for the AHHA trial in which villages (rather than households) were assigned to treatment would likely reduce but not completely avoid contamination of treatments between trial arms.

No participants expected to receive free flour as part of any food distribution programme over the next 2–3 months.

The majority of participants stored their maize flour in sacks (n = 39), while others reported using plastic buckets and cloths.

No participants reported using at-home fortification or taking micronutrient supplements. Only one participant reported use of micronutrient supplements in their household (vitamin A, by a child aged 5–10 years).

### Focus group discussions and key informant interviews

Three FGDs were conducted with adult men and three FGDs with adult women. In addition, seven in-depth KIIs were completed. Following transcription of FGDs and KIIs, a code tree was developed, and quotes were categorised (Table 3).

**Table 3:** Focus group discussion and key informant interview code tree

Main category	General category	Sub-category
Flour consumption	Flour type preferences	
	Location of flour consumption	
	Sharing / gifting / selling maize flour	
Intervention perception	Trust in actual maize meal content	Family planning tool, Benefits from the intervention
	Trust in blood sampling	Actual purpose of blood sampling, Amount of blood sample, Who is going to draw blood samples and where
How recipients/participants are perceived by wider community	Jealousy	Name calling (“Lazy”) Stigma
Community sensitisation	The need for sensitisation	
	The process of sensitisation	

### Flour consumption

The most appropriate type of flour to distribute was explored in FGDs and KIIs. Participants noted:

*“It should be granmill because you can cook porridge as well.”* (FGD F1)

*“Granmill is okay because it will cater for all the meals.”* (FGD F1)

The potential issue of households eating maize flour outside the home was also explored in the FGDs and KIIs. Participants suggested that eating at home as a family unit is most common:

*“These days each family eats on their own.”* (FGD M3)

*“It’s rarely that we share with our neighbours.”* (FGD F3)

*“The eating of food from other houses cannot be much. Maybe not very often, it can be once or twice a month, it’s not every day…it is different from the past. In the past, women used to come together, several households come together with plates of food. But nowadays things are changing, so the owner of this house is supposed to eat in this house.”* (KII, Agricultural extension worker)

Participants suggested that, if they were receiving maize flour as part of a trial, they could give their own flour instead of the study flour for special occasions such as weddings, funerals or church gatherings:

*“We will get the flour from our fields that we have kept.”* (FGD F1)

*“We cannot share [the trial flour] because this will be specific for a purpose. Probably we can give the maize flour that we grow*.” (FGD M1)

Several FGD participants raised the issue of guests, and that it would not be culturally appropriate to cook a different type of flour for guests:

*“It is wrong to cook and eat for the family and prepare another for the visitor. The visitor will think we have poisoned the food. The visitor will not be comfortable to eat the nsima unless we eat together.”* (FGD F2)

*“…when we have the visitor, they will have to eat the same flour.”* (FGD F1)

There was a risk that participants would simply sell the trial flour. However, the FGD participants noted:

*“Selling things that you have been given it’s not good. We sell things that we have worked for.”* (FGD M1)

*“It’s better we sell what is ours, than what we are receiving because that may compromise the study.”* (FGD F1)

The qualitative results supported the provision of maize flour rather than maize grain because maize flour is less likely to be sold than grain.

*“…people here will be surprised to see you selling the flour. It’s in town where we see people selling flour.”* (FGD M2)

*“The only year when I saw people selling maize flour was the year when there was hunger the whole country. Then people were indeed selling maize flour. There’s no one who can bring maize flour on the market and expect people to buy.”* (FGD M3)

However, distribution of flour rather than grain would not eliminate the risk of selling:

*“It’s better off to give them flour unlike the actual grains*, *but still we should anticipate some maybe going that far of selling, because I have seen even some people selling the flour they have gotten from the hospital, the therapeutic flour for their child under the CMAM [Community Management of Acute Malnutrition] programme.”* (KII, District-level Nutrition Officer)

*“They [the men who sell flour] put the flour in their trousers and when they get to the beer hall, they exchange with beer.”* (FGD F3)

*“Sometimes it is us women, we have a problem, if our neighbour has no maize flour and she has money, we steal from our house and sell the maize, while the husband is away.”* (FGD F3)

*“The maize flour we put in a bucket when we are going to draw water. Our husband thinks the bucket is empty yet there is maize flour which we are going to sell at the borehole to another woman.”* (FGD F3)

Involving the village chiefs in the process would potentially reduce risk of selling flour:

*“In our village it is not possible [to sell maize] because when we harvest, we are called by the chief who addresses us. He advises us not to sell the maize because, like this year we did not harvest well so he would tell us not to sell the maize and to care for the food. Because of that we do not sell.”* (FGD F3)

### Intervention perception

Focus group discussion findings revealed the participants had mixed perceptions of the proposed trial. Most community members perceived potential benefits and motivation of the intervention (i.e., the receipt of free maize flour for 10 weeks).

*“We will eat the trial maize flour because we don’t have adequate maize flour in our houses”.* (FGD F2)

*“They will give us free maize flour and we will know our nutritional status after blood test”*. (FGD M1)

Similar observations were made by key informants:

*“The motivation [to participate] is food. If you give them food, they will be happy. That’s the motivation.”* (KII, Agricultural extension officer)

Participation in previous research also seemed to influence their perception of the proposed trial:

*“Most of the farmers have positive experience with research because they know that after the research those things will be implemented for their own benefit, so they welcome that*.” (KII, Agricultural extension officer)

On the other hand, some participants had reservations. The main areas of concern were (i) lack of trust in what will be added to the AHHA trial maize or maize meal, (ii) fears over the blood sampling routine, and (iii) concerns regarding negative social stigma.

Community perceptions of blood sampling were critical, because blood samples would be required in the AHHA trial to assess the intervention’s impact on selenium status. Regarding blood sampling, some respondents felt that sampled blood might be used to assess the prevalence of HIV in the area, whereas others were concerned about the amount of blood that would be collected. One of the respondents said:

“*How much blood will be collected? Won’t they test other diseases*?” (FGD M1)

Another respondent said:

“We *will provide blood samples once we know the required amount.*” (FGD F2)

Other concerns related to whether the blood samples would be used for non-health–related purposes. One of the respondents said:

“*Maybe blood sampling has to do with Satanism*.” (FGD F2)

Another participant said:

“*Once the child is sick, many will say; you are in the camp of Satanist; they fed you well to become fat so that they can kill you*.” (FGD F3)

On the other hand, most respondents indicated they were not concerned with the sampling of blood, as illustrated by the following quote:

“*Blood sampling will not be a problem since you said you will only test nutrients in the blood.*” (FGD M1)

Another respondent said:

“*Blood sampling will not be a problem since small amount of blood will be sampled.*” (FGD M3)

Community perceptions of the quality/safety of the flour and the researchers’ intent in providing trial flour is critical because participants and other household members must be happy to consume the allocated flour. One of the major obstacles noted by participants was trust in the trial flour.

*“…people will relate to anything they receive to be not real things, they will think that it is sort of fake”* (KII, District-level Nutrition Officer)

Male (but not female) FGD respondents identified the risk that maize meal would be perceived as a family planning tool. In response to whether children would be allowed to eat the maize meal enriched with selenium, one respondent said:

“*If the maize meal can make our children infertile then children will not be allowed to eat. But if it will make them healthy as indicated, then children will eat since they eat what parents eat.”* (FGD M3)

The observation was also noted by key informants:

*“…they might think you are involving them in family planning.”* (KII, Health Surveillance Assistant)

Previous health-related programmes have identified similar issues when free items were distributed in the community:

*“I will give you an example of the mosquito nets. So, government distributes mosquito nets to the people, and then the people say that these mosquito nets are there to make them…not to make them have babies…they affect fertility.”* (KII, District-level Information Officer)

Micronutrient programmes in Kasungu District have faced similar challenges:

*“People were saying just don’t go to the hospital because they have asked you to go…don’t just give your children vitamin A, which is aimed at making future generations impotent.”* (District-level Nutrition Officer)

Despite these concerns, most respondents indicated that they would be willing to eat the maize meal provided if it was endorsed by government officials:

“*Other people will eat the maize meal because they trust the recommendations by government*.” (FGD F1)

“*We can eat the maize meal because we feel it is good for us since the government has approved it*.” (FGD F1)

Previous food distribution schemes caused upset in communities because so few households benefitted:

*“There’s been an outcry of course, and I remember at one point in time, the chiefs could not even manage to allow the partner to distribute the food, just because it has come little compared to the number that is supposed to benefit from that.”* (KII, NGO Nutrition Advisor)

When it was proposed that everyone will be receiving the flour, including the village chief, participants reported that this would likely reduce fear and suspicions:

*“This is something very good because there will be no people complaining. If that is the case, then most people will be consuming the flour. Because they know that each and every one is consuming this flour. It’s not strange flour.”* (KII, Agricultural Extension Officer)

The timing of the flour distribution was also discussed:

*“If you change the months, maybe starting in the lean period, it will also be taken as relief food.”* (KII, District Information Officer)

### How recipients/participants are perceived by wider community

Concerns were raised about the social stigma associated with receiving free food. Some community members felt people outside the study area would stigmatise the beneficiaries (partly because of jealousy):

“*Community members talk a lot about free food. Sometime back, people received free maize; they nicknamed them as lazy people.*” (FGD M1)

A similar observation was noted by key informants based on their experience of food distribution programmes:

*“Of course, the ones who were not receiving [food distributions], they were maybe just jealous…so that’s why they were calling them ‘Manjalende’ [lazy people who can’t feed themselves]*.” (KII, District-level Nutrition Officer)

And:

*“[Those who did not receive the food distribution] were calling them ‘Manjalende’, it’s like the people were just waiting for free food.”* (KII, District-level Information Officer)

However, this concern was not universally shared:

*“…the neighbouring village would be talking, saying ‘oh those people they are very lucky’… they cannot talk negatively, this is food. They would just admire. They can just appreciate that their friends are very lucky.”* (KII, Health Surveillance Assistant)

### Community sensitisation

The results of the formative research underscored the importance of community sensitisation. Good sensitisation—appropriate communication conducted by the right people—would be essential to overcome negative perceptions.

*“…every new thing it’s received with some doubts. People talk because there’s no information. If people have information and have everything, it clears out those misconceptions.”* (KII, District Information Office)

Sensitisation would be important to garner community support for the trial:

*“The first thing is sensitisation. You can sensitise them, there after we shall be working hand-in-hand together with them.”* (KII, School head teacher)

With good sensitisation, participants would be more likely to consume the flour:

*“I don’t think rejecting [the flour] can happen, first of all there has to be strong community mobilisation, people they have to be sensitised fully, why that is being done.”* (KII, District-level Nutrition Officer)

Sensitisation was important to address negative perceptions and fears related to blood sampling.

*“They can’t raise issues if people can be sensitised before…They must know that our friends are going there for this, we were told about this, so that’s why these women are going over there.”* (KII, Health Surveillance Assistant)

*“…because blood sometimes can be a delicate thing in our African culture, but if they understand why you are doing that, I don’t think there can be a problem.”* (KII, District-level Nutrition Officer)

Several key informants noted the importance of involving Health Surveillance Assistants in the sensitisation process. Their support was important to gain trust in blood collection:

*“…people are [used to] giving blood samples to detect malaria. You know the Health Surveillance Assistants, they go and collect samples of blood… so the HSAs should be there to tell the people and answer their questions.”* (KII, Agricultural Extension Officer)

The health surveillance assistants (HSAs) also provide an important point of contact with the communities, helping to direct sensitisation efforts and addressing particular concerns:

*“The HSA is the one who works directly with the communities… I think the HSA is the first person who gets information from the household.”* (KII, District-level Information Officer)

The other key individuals identified for the sensitisation process were those in positions of authority or influence:

*“…the chiefs, they are also the ones who can help, because they respect them, so what the chiefs may say to the villagers, the villagers understand very well that our chief is supporting this.”* (KII, District-level Nutrition Officer).

*“The church has to be involved because a large number of people gather there, so it’s very easy to distribute the information.”* (KII, Health Surveillance Assistant)

An important lesson from the gender segregated FGDs was that men, in this patrilocal society, placed more trust in their village headman, whereas women placed more trust in the HSAs with whom they had frequent interactions through antenatal clinics.

“*The message on feeding trials will be underrated (anthu akanyozera), if it is disseminated by the village headman alone.”* (FGD F1)

Participants noted the importance of involving groups who can represent the community during the sensitisation process. The community care group volunteers were cited as an appropriate network:

“*They [the community care group volunteers] are the first people I would use…to disseminate information.”* (KII, Health Surveillance Assistant).

## Discussion

The formative research provided valuable information on the feasibility of the AHHA study and guided subsequent decisions on the AHHA trial design and implementation, as follows:

Prior to the formative research, two possible designs were being considered: first, a design where households are randomly allocated to treatment and, second, a cluster-randomised design with villages randomised to treatment. The first design would only be appropriate if households did not share the trial flour with other households. If sharing were frequent and unavoidable, a cluster-randomised design (e.g., at village level) might be more appropriate since this might reduce ‘contamination’ between treatments; however, a cluster-randomised trial would necessitate a larger sample size than an individually randomised design, which is disadvantageous for ethical and resourcing reasons.

Based on the findings from the formative research, it was decided that randomisation of treatment at the household level would be feasible and appropriate. To reduce the risk of contamination through sharing of flour, we would clearly explain the need to consume the AHHA trial flour at home and to source gifts from their own flour production. To minimise sharing, we decided to provide flour to all households in the study area, not only to those participating in the trial. Specifically, we would purchase control-equivalent, non-biofortified flour and provide it to non-trial households in the same quantities and at the same distribution time points as the trial households. Providing flour to non-participating households would also help disassociate the trial from food distribution programmes that target assistance to impoverished households, which would reduce potential negative social stigma.

Additionally, the timing of the flour distribution was chosen to avoid the ‘lean’ or ‘hungry’ season (i.e. December–March), when food aid programmes typically distribute relief food.

The formative research findings informed the decision to distribute flour rather than grain, mainly to reduce the risk of selling. The type of flour selected was *granmill* due to participants reporting this as their preference for gifts. As found in the FGDs, their preference was likely due to the ability to use *granmill* for porridge (typically consumed at breakfast) in addition to *nsima* (typically consumed at lunch or dinner). This was advantageous from the trial perspective as it would likely increase adherence.

To reduce the risk of running out of flour and to allow households to cater for guests, a decision was made to provide slightly more flour than was required to meet a typical family’s energy needs. Specifically, 330 g *capita*^-1^ day^-1^ of maize flour was provided for all family members including for children, which was rounded up to the nearest 5 kg at each 2-week distribution time point.

Selling and sharing of flour was a risk, so it was decided to monitor adherence every 2 weeks among participant households, and to discourage selling and sharing of AHHA trial maize flour through community engagement.

Following the formative research findings, trial sensitisation activities were conducted from ~ 8 months prior to recruitment until completion of the endline survey. The formative research findings influenced the decision on which community members to engage in the sensitisation (including HSAs and village headmen), and how best to work with them to conduct community briefings and disseminate information about the trial. Based on the formative research findings, the sensitisation activities also reached villages neighbouring the study area to clearly explain what the trial involved and why they were not selected to participate.

To help address any potential mistrust in the flour, the trial team arranged for community members from participating villages to visit the maize production site during crop production, and at a later date to view the milling and packaging process. A community tasting session was also conducted in the study area prior to the first distribution of flour, with community members and those involved in sensitisation invited to participate. These activities all increased trust in the flour and mitigated risks of negative rumours. In addition, the trial maize flour was packaged in sacks similar to those used for packaging commercial maize meal. These sacks were clearly labelled with the logo for Lilongwe University of Agriculture and Natural Resources, the lead implementing partner.

To ensure trust in blood sampling, the project sensitised the community regarding the amount of blood to be collected at baseline and endline surveys (6 ml) and nurses from the Ministry of Health were recruited to collect the samples. During recruitment for the AHHA trial, research assistants took with them example blood collection vials to demonstrate the volume of blood that would be required. Mobile, tented clinics were set up in public places, which had been identified though community consultation; this was deemed less likely to raise suspicions related to witchcraft compared with collecting blood samples in private houses. The community members were informed that blood samples would only be used to assess micronutrient status, and HIV/other disease status would not be measured.

Sensitisation continued throughout the survey and intervention periods, with members of the trial team engaging regularly with the community, extension workers and volunteers to answer questions, allay concerns or address negative misconceptions. Establishing close and trusted bonds with communities is recognised as an important process for ensuring the successful and ethical conduct of community-based trials [13], and was very important to the successful implementation of the AHHA trial. Similar findings have been reported in formative research conducted in preparation for a randomised control trial to test biofortified wheat flour, conducted in Pakistan [12]. Community engagement will continue now that the trial is complete, with results of the trial communicated to participants and the wider community.

Strengths of the present study included the mixed-methods approach with questionnaire, FGD and KII components, which gave the study team a rich understanding of potential challenges in delivery of the AHHA trial as well as valuable guidance on addressing these. The separation of male and female FGDs was valuable, and identification of KIs through snowball sampling provided an effective way to gather input from a variety of sources. Conducting the research in the local language with trained research assistants was essential. Finally, conducting the formative research ~ 12 months prior to the implementation of the trial provided sufficient time to incorporate learnings into decisions on trial delivery and design, including the initiation of sensitisation activities ~ 8 months prior to participant recruitment.

Limitations of the study include the relatively small number of FGDs, although the study team found that thematic saturation was reached. The formative research was conducted near to the AHHA study area, with the aim of providing a relevant population while avoiding influencing potential trial participants. However, it was possible that the views and perceptions of participants in the formative research would differ from participants in the AHHA trial.

## Conclusion

The formative research findings provided valuable information on the feasibility of the AHHA trial, a community-based randomised, controlled trial conducted in rural Malawi, and the findings were used to inform and guide the design and delivery of the trial. The formative research was essential to inform the community sensitisation process for the AHHA trial, since participant fears could be anticipated (e.g. that the flour might affect fertility or that blood sampling may be linked to witchcraft) and the appropriate local agents could be engaged for sensitisation activities (e.g. agriculture and health extension workers, village head men, religious leaders and teachers).

## Supplementary Information

**Additional file 1.** Household survey questionnaire

**Additional file 2.** Focus Group Discussions: Facilitator’s manual

**Additional file 3.** Structured Interview for Key Informants

**Additional file 4.** The TIDieR (Template for Intervention Description and Replication) Checklist*

## Data Availability

The datasets used and/or analysed during the current study are available from the corresponding author on reasonable request.

## Abbreviations

AHHA: Addressing Hidden Hunger with Agronomy
EPA: Extension planning area
FGD: Focus group discussion
HIV: Human immunodeficiency virus
HSA: Health surveillance assistant
KII: Key informant interview
TA: Traditional authority
