# Supplementary material for: Preparing for a community-based agriculture-to-nutrition trial in rural Malawi: formative research to assess feasibility and inform design and implementation decisions
Source: Pilot Feasibility Stud. 2021 Jul 7;7:141. doi: 10.1186/s40814-021-00877-1 (PMC8262007; doi:10.1186/s40814-021-00877-1)
Supplement: Supplementary file 1 — Additional file 1. Household survey questionnaire [file 40814_2021_877_MOESM1_ESM.docx]

### Additional File 1. Household survey questionnaire

| 0 | I’d like to ask you to describe everything that you ate or drank yesterday during the day or night, whether at home or anywhere else. | Introduction |
| --- | --- | --- |
| 1.1 | Did you eat breakfast yesterday? | Yes/No |
| 1.2 | What food items did you eat for breakfast? Proceed until respondent cannot recall any further food items or dishes. | Free text |
| 1.3 | Did you eat breakfast from a shared plate? | Yes/No |
| 1.4 | What dish (or dishes) did you share at breakfast? | Free text |
| 2.1 | Did you eat lunch yesterday | Yes/No |
| 2.2 | What food items did you eat for lunch? Proceed until respondent cannot recall any further food items or dishes. | Free text |
| 2.3 | Did you eat lunch from a shared plate? | Yes/No |
| 2.4 | What dish (or dishes) did you share at lunch? | Free text |
| 3.1 | Did you eat dinner yesterday? | Yes/No |
| 3.2 | What food items did you eat for dinner? | Free text |
| 3.3 | Did you eat dinner from a shared plate? | Yes/No |
| 3.4 | What dish (or dishes) did you share at dinner? | Free text |
| 4 | Write any observations on yesterday's consumption, for example, why they missed eating meals? | Free text |
| 5.1 | Yesterday, did you eat nsima or maize porridge prepared outside the home? | Yes/No |
| 5.2 | Where did you eat the nsima/maize porridge? | Close neighbour (within 10 metres)  Other household within the village  Household outside the village  Restaurant/other |
| 5.3 | Was there a special occasion, such as a wedding? | Yes/No |
| 5.4 | What was the special occasion? | Free text |
| 6.1 | Yesterday, did any guests eat nsima or maize porridge prepared at your home? | Yes/No |
| 6.2 | For each guest visit, where do they live? | Close neighbour (within 10 metres)  Other household within the village  Household outside the village |
| 7.1 | Yesterday, did you give or sell any maize flour to another household? | Yes/No |
| 7.2 | For each occasion, how much flour did you give? Estimate in kg | Less than 1 kg  1-5 kg  5-20 kg  >20 kg |
| 7.3 | For each occasion, where did the recipient live? | Close neighbour (within 10 metres)  Other household within the village  Household outside the village |
| 8.1 | Yesterday, did you receive any maize flour from another household, either as a purchase or a gift? | Yes/No |
| 8.2 | For each occasion, how much flour did you receive? Estimate in kg | Less than 1 kg  1-5 kg  5-20 kg  >20 kg |
| 8.3 | For each occasion, where is the household from which you bought or received maize flour? | Close neighbour (within 10 metres)  Other household within the village  Household outside the village |
| 9.1 | What type/types of maize flour do you plan to consume at home over the next month? | Mgaiwa (unrefined)  Granmill (partially refined)  Ufa woyera (refined)  Madea |
| 9.2 | If you were gifted maize flour for the next month, what type of maize flour would you prefer? 1 = most preferred, 4 = least preferred | Mgaiwa (unrefined)  Granmill (partially refined)  Ufa woyera (refined)  Madea |
| 9.3 | Write any observations on flour preferences, including why preferences might differ from planned consumption? | Free text |
| 10.1 | Do you expect to receive maize flour through any food distribution programme in the next 2-3 months? | Yes/No |
| 10.2 | Through what scheme do you expect to receive maize flour? | Free text |
| 11.1 | What material or container do you use to store your maize flour? | Free text |
| 11.2 | Any relevant observation on material/container | Free text |
| 12.1 | Does your household use any at-home micronutrient fortification, such as sprinkles? | Yes/No |
| 12.2 | What micronutrient at-home fortification does your household use? Note down the actual micronutrients in the fortificant | Free text |
| 12.3 | Do you use any micronutrient supplement? | Yes/No |
| 12.4 | What micronutrient supplementation do you use? Note down the actual micronutrients in the supplement. | Free text |
| 12.5 | Do your children aged 5-10 years use any micronutrient supplement? | Yes/No |
| 12.6 | What micronutrient supplementation do they use? Note down the actual micronutrients in the supplement. | Free text |
