# Supplementary material for: Preparing for a community-based agriculture-to-nutrition trial in rural Malawi: formative research to assess feasibility and inform design and implementation decisions
Source: Pilot Feasibility Stud. 2021 Jul 7;7:141. doi: 10.1186/s40814-021-00877-1 (PMC8262007; doi:10.1186/s40814-021-00877-1)
Supplement: Supplementary file 2 — Additional file 2. Focus Group Discussions: Facilitator’s manual [file 40814_2021_877_MOESM2_ESM.docx]

### Additional File 2. Focus Group Discussions: Facilitator’s manual

The questions below are indicative of the discussion route. The exact questions may deviate based on the expertise of the FGD facilitator.

**Questions**

**“Warm up” question**

1. What are your favourite dishes?

**Decision making and nutritional knowledge**

1. Who decides what you eat each day? Is it you or another family member?
2. Do you consider your diets are nutritionally adequate?
3. Where do you get information on nutrition and health?

**Perceptions of the proposed feeding study***. Explain that the study team is preparing to conduct further research in the community. The study will involve all members of the village receiving flour every 2 weeks for 12 weeks. The amount of flour will be sufficient for all members of the household to consume 3 meals a day.*

1. If you were to receive flour every two weeks for twelve weeks, would you eat it?
2. What else might you do with the flour?

***Explain that some of the households will receive flour that was enriched with micronutrients during crop production.***

1. Would you have any concerns about eating this flour, or preparing it for your children?

**Explain that a random selection of households within the village will need to provide blood samples before the feeding study and at the end of the 12-week feeding study.** *This would involve collecting a small amount of blood from an adult woman and a school aged child (5-10 years of age).*

1. Would you be comfortable providing a blood samples?
2. And what about your child, would you be happy for them to provide blood samples?
3. Would you have any fears or concerns about giving blood samples?

**Then move on to general questions about the feeding study:**

1. What advantages do you see in participating in the proposed feeding study?
2. What fears or concerns would you have about participating in the study?
3. What information would you like to know before participating in the proposed feeding study?
4. How would you like to receive information on the study? Through written information sheets, through live question and answer sessions, etc.?
5. Which figures of authority or trust would you like to discuss with, prior to enrolling in the feeding study?

**Managing potential problems***. Explain that a high level of compliance (i.e. exclusively consuming flour allocated to your household) is required for the trial to work.*

1. If you were provided with a 25 kg bag of flour as part of the study, what would you do with it? [Facilitator may prompt: sell the flour, share with neighbours, etc.]
2. Would you have any concerns or suspicions about eating the flour or using it to feed your children?
3. What information could the study team provide to encourage compliance?
4. Which individuals or institutions in your community would you like to consult about your concerns or suspicions?
5. Would you be willing to modify your behaviour to increase your compliance, i.e. restrict the frequency with which you eat outside the home?
6. What would you do if a neighbour or relative complained that the feeding study flour was harmful?

**Past experiences**

1. Have you been involved in a research study before? What was your experience of participating?
2. Have you received flour as part of a food distribution scheme before? What was your experience of this?

**Wrap-up**

1. All things considered, would you welcome the implementation of the proposed feeding study in your village?
2. If you were to explain what we discussed to a neighbour or relative, what would you say?

**Provide a brief oral summary, then ask:**

1. Is this an adequate summary?

**Briefly review the purpose of the study, then ask:**

1. Have we missed anything?

**Thank the participants for their time and inputs. Explain what happens next in terms of preparations for the feeding study*.***
