# Supplementary material for: Preparing for a community-based agriculture-to-nutrition trial in rural Malawi: formative research to assess feasibility and inform design and implementation decisions
Source: Pilot Feasibility Stud. 2021 Jul 7;7:141. doi: 10.1186/s40814-021-00877-1 (PMC8262007; doi:10.1186/s40814-021-00877-1)
Supplement: Supplementary file 3 — Additional file 3. Structured Interview for Key Informants [file 40814_2021_877_MOESM3_ESM.docx]

### Additional File 3. Structured Interview for Key Informants

| i | Date |  |
| --- | --- | --- |
| ii | Time |  |
| iii | Interviewer ID |  |
| iv | Key Informant ID |  |
| v | Respondent has provided consent? |  |
| 1 | How would you describe the job you do and your role in the community? |  |
| 2 | Tell me about any of research studies in your community? Were you involved and what was your role? |  |
| 3 | What were the community’s perceptions of the research study? Were there any challenges? What could have improved the interaction between the researchers and the community? |  |
| 4 | Tell me about any food distribution programmes in your community? Were you involved and what was your role? |  |
| 5 | What were the community’s perceptions of the food distribution programme? Were there any challenges? What could have improved the interaction between the food distributors and the community? |  |
| 6 | [INTERVIEWER EXPLAINS THE PROPOSED FEEDING STUDY]  Would you welcome the involvement of your community in the proposed feeding study? |  |
| 7 | What advantages would you hope the study brings? |  |
| 8 | What concerns would you have for the wellbeing of your community? |  |
| 9 | What information would you like to know before supporting the proposed feeding study? |  |
| 10 | With whom would you like to discuss the proposed feeding study? |  |
| 11 | Which individuals or organisations should be involved in the feeding study sensitisation process? |  |
| 12 | Which key informants should we consult as we prepare for the feeding study? |  |
